# Mobile phone use and risk for intracranial tumors

**DOI:** 10.1186/s12952-015-0043-7

**Published:** 2015-12-23

**Authors:** George A. Alexiou, Chrissa Sioka

**Affiliations:** Neurosurgical Institute, University of Ioannina, Po box 103, Neohoropoulo, Ioannina 45500 Greece

## Abstract

Mobile phone use has been discussed over the last few decades with increased risk for intracranial tumors. The majority of studies have been conducted on gliomas and meningiomas. Although some case-control studies have found a positive association between the use of mobile phones and the risk of tumors, other studies have reported no significant association. A possible long-term mobile phone use may lead to increased risk however, the evidences are not yet conclusive and further studies are needed. In the present study we reviewed the current evidence for the association between mobile phone use and risk for intracranial tumors.

## Background

Over the last three decades an increasing use of mobile phones is evident worldwide. More than one billion of mobile phone users have been reported to exist. The increasing use of mobile phones has raised concerns of health risk and especially for intracranial tumors, since brain is the nearest organ than is in close contact with the radiofrequency electromagnetic fields, emitted by mobile phones. An even greater increased risk has been suggested for children due to thinner skull, smaller head and increased brain conductivity [1]. Nevertheless, other studies have not verified this risk [2].

Apart from mobile use, cordless phones may be potentially linked to increased risk of brain tumor. On 2011 the WHO International Agency for Research on Cancer categorized radiofrequency electromagnetic fields from mobile phones, and from other devices, as a Group 2B, a possible human carcinogen [3, 4]. Investigation of the effects which RF may produce in cellular level in vivo and in vitro revealed increased risk of cell death and cancer development in mice. The potential effect of RF to germ cells is worrisome since it can be transmitted to subsequent generations [5]. Nevertheless, a meta-analysis of the available data on the genetic damage in human cells exposed to non-ionizing radiofrequency fields revealed no significant effect [6].

Among intracranial brain tumors in adults, meningiomas are the most common, accounting for 36 % of all, followed by gliomas (28 %). Glioblastoma (WHO Grade IV) is the most malignant primaty brain tumor, accounting for 15.7 % of all tumors. Other types are pituitary adenomas (13.1 %), other neuroepithelial (5.1 %), lymphoma (2.4 %), oligodendrogliomas (2 %), ependymomas (1.8 %), embryonal tumors (1 %), craniopharyngioma (0.7 %) and acoustic neuromas (0.6 %) [7]. Regarding pediatric brain tumors, pilocytic astrocytoma is the most common tumor, followed by medulloblastoma and ependymoma [8]. In the present study we reviewed the current evidence on the association between mobile phone use and risk for intracranial tumor.

## Material and methods

### Criteria for study eligibility

We considered all English-language studies providing original data on the association of mobile phone use and risk for intracranial tumor that were published over the last decade. We focused mainly on gliomas, meningiomas, acoustic neuromas and pituitary tumors, since few evidence for other tumors exist [9]. All study designs were eligible. We excluded articles with non original data and duplicate publications.

### Search strategy for identification of studies

PubMed searches were performed using a wide array of terms pertinent to mobile phone use and intracranial tumor. The exact search (last updated in September 2015) is available from the authors upon request. Moreover, the reference lists of eligible articles and pertinent reviews were scrutinized. Retrieved articles were evaluated for eligibility by two independent investigators and disagreements were solved by consensus. From each eligible study, we extracted the following information: author; journal; year; design; study population and details on the definitions of all reported analyses and their reported statistical significance.

## Results

### Description of studies

**Fig. 1:**
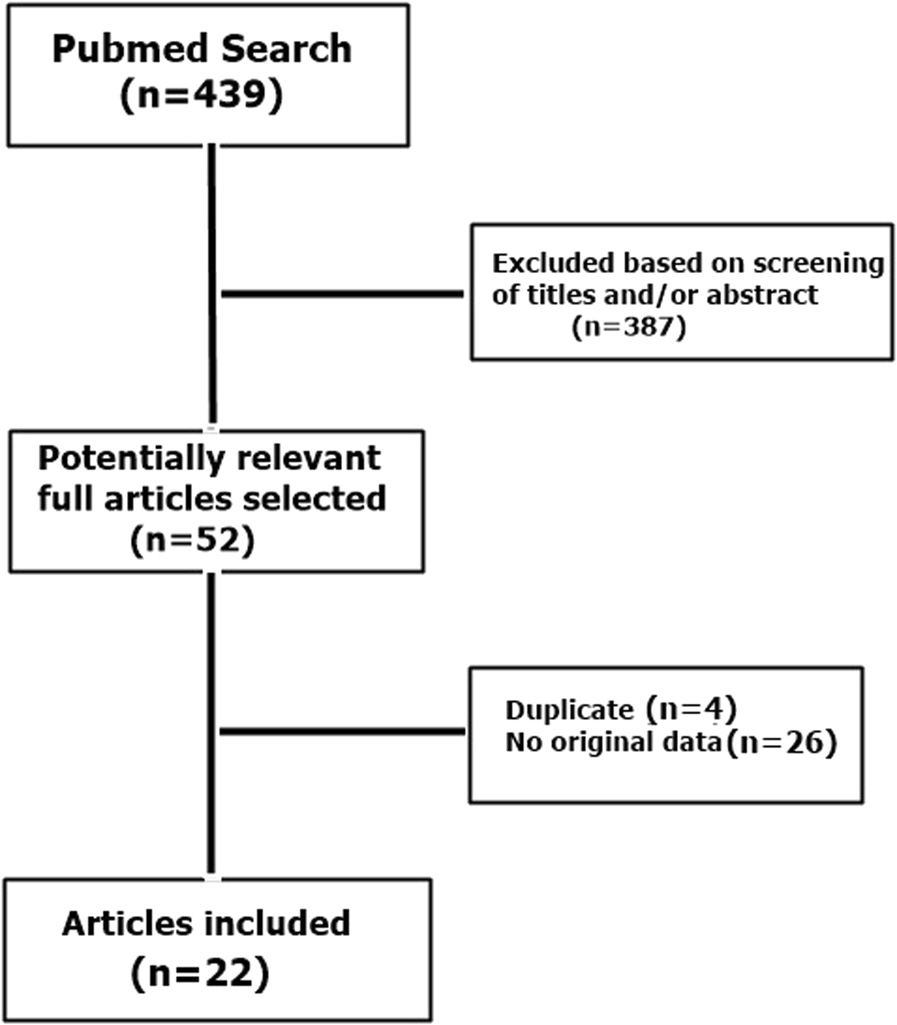
Flow chart of study selection

The electronic literature search yielded 439 items. Of those, 387 were irrelevant to the project based on examination of the title and abstract, while 52 articles were relevant or their relevance could not be decided upon examining the title and abstract. The 52 articles were examined in full-text and 22 met the inclusion eligibility criteria (Fig. 1) [2, 9–29]. There were two cohort studies of mobile phone users and 20 case-control studies on this topic. Reasons for exclusion were no original data (*n* = 26) and duplicate/overlapping data (*n* = 4).

### Gliomas

Several studies have investigated whether mobile phone use is associated with an increased risk for gliomas [10–16]. Nevertheless, the results are still not conclusive. A recent study analysis of two case-control studies, on malignant brain tumors, that included 1498 cases and 3530 controls, revealed that mobile phone use increased the risk of glioma. The risk was nearly double in the group with over 25 years latency period. An increased risk was also found for the cordless phone use. Furthermore, in the same study it was of note the finding that temporal lobe had the highest risk for glioma occurrence [17]. Another case-control study, conducted in France, that included 253 gliomas, 194 meningiomas and 892 matched controls, revealed no association between mobile phone use and risk for gliomas. Nevertheless, in heavy mobile phone users with cumulative duration over 896 h or number of calls over 18,600 there was a significant increased risk for glioma [14]. A decreased survival of glioma patients with long-term use of wireless phones has been also recently reported [30]. In low grade gliomas, although mobile phone use has been associated with increased risk, when focusing on survival, a survival benefit was reported in low grade glioma patients with mobile phone use. The authors hypothesis was that tumor volume was larger in exposed than in unexposed patients, which would permit an earlier diagnosis and surgical intervention [30]. In 2010 Hardell et al reported an increased risk for glioma for both short and long term mobile phone users. Nevertheless, one possibly bias of this study was that for deceased patients, data on exposure were collected from relatives up to 11 years after death [19]. Hardell et al included patients 20–80 years [19]. This is important since the highest incidence of glioblastoma, the most common and malignant brain tumor, is found in the age group 45–75 years.

The largest study conducted to date was the INTERPHONE study, which was conducted in 13 countries with 16 centers. This was an interview-based case-control study and involved 2409 meningioma, 2708 glioma cases and matched controls. The studies included patients 30–59 years. The results showed the absence of increased risk of glioma with use of mobile phones. There was suspicious of an increased risk of glioma at the highest exposure levels, but further investigation is needed in order to draw safe conclusions [20]. It is of note that the overall ORs in some of the included studies were <1.0, suggesting possible methodological drawbacks. In fact in the studies included in the INTERPHONE study no blinding was used. Finally a large prospective study that investigated the association of mobile phone use and incidence of intracranial tumors and other cancers in 791,710 middle-aged women in UK found no appreciable association for glioma or meningioma [28]. A Danish cohort study that included 358,403 subscription holders accrued 3.8 million person years found no increased risk for glioma even for individuals with more than 13 years of subscription [29]. Other older studies also showed no strong relation between mobile phone use and gliomas (Table 1) [10–16].

**Table 1:** Odds ratios (ORs) and 95 % confidence intervals (CIs) from case–control studies on gliomas

Study	No of Cases	No of Control	OR (95 % CI)
Hardell et al. 2015 [17]	1.498	3.530	1.3 (1.1–1.6)
Coureau et al. 2014 [14]	253	892	1.24 (0.86–1.77)
Hardell et al. 2013 [15]	593	1368	1.6 (0.99–2.77)
Aydin et al. 2011 [2]	352	646	1.36 (0.92–2.20)
Cardis et al. 2011 [16]	553	1.762	0.93 (0.73–1.18)
Hardell et al. 2010 [19]	346	276	2.4 (1.4–4.1)
INTERPHONE Study 2010 [20]	2.708	2.972	0.81 (0.70–0.94)
Klaeboe et al. 2007 [10]	96	227	0.8 (0.5–1.1)
Takebayashi et al. 2008 [23]	88	118	0.7 (0.4–1.2)
Hepworth et al. 2006 [31]	966	1.717	0.94 (0.78–1.13)
Christensen et al. 2005 [12]	83	193	0.66 (0.46–0.95)
Lönn et al. 2005 [13]	371	674	0.73 (0.55–0.96)

#### Meningiomas

In meningiomas 8 case control studies were identified (Table 2) [9–11, 13, 14, 16, 18, 20]. A pooled analysis of two Swedish case-control studies on 1625 meningioma patients and 3530 control patients showed a relative increased risk (OR = 1.2, CI = 0.9–1.6) among heavy users of mobile and cordless phones [18]. The INTERPHONE study that has been analyzed previously, reported no increased risk of meningioma in individuals that used a mobile phone [20]. Similarly, in the Danish cohort study, among those with mobile phone subscriptions of over 10 years, the risk ratios were 0.90 (0.57 to 1.42) in men and 0.93 (0.46 to 1.87) in women for meningioma [29]. Further evidence on absence of association between meningioma occurrence and mobile phone use have been provided by the study in five North European countries [10]. The results showed no increased risk in relation to years since first use, lifetime years of use, cumulative hours of use or cumulative number of calls [10]. Since meningioma is a slow-growing tumor, longer latency period is obviously needed in order to draw definitive conclusions.

**Table 2:** Odds ratios (ORs) and 95 % confidence intervals (CIs) from case-control studies on meningiomas

Study	No of Cases	No of Control	OR (95 % CI)
Carlberg et al. 2015 [18]	1.625	3.530	1.2 (0.9–1.6)
Coureau et al. 2014 [14]	194	892	0.90 (0.61–1.34)
Cardis et al. 2011 [16]	676	1.911	0.80 (0.66–0.96)
INTERPHONE Study 2010 [20]	2.409	2.662	0.83 (0.61–1.14)
Lahkola et al. 2008 [9]	1.204	2.945	0.76 (0.65–0.89)
Takebayashi et al., 2008 [23]	132	229	0.70 (0.42–1.16)
Klaeboe et al. 2007 [10]	207	358	1.2 (0.6–2.2)
Lönn et al. 2005 [13]	273	674	0.7 (0.5–0.9)

#### Acoustic neuroma

Acoustic neuromas are also slow-growing tumors, thus observation period should not be short. For acoustic neuroma, a large prospective study reported an increased risk with long term use compared to patients who never used a mobile phone. Furthermore, the risk increased with increased duration of use [28]. The results of the INTERPHONE study on acoustic neuroma showed no increased risk in the first decade after starting mobile phone use [21]. Risk of a tumor on the same side of the head as the reported phone use was increased only for use over 10 years (OR = 1.8, 95 % CI: 1.1–3.1) [21]. In a population-based case-control study in Germany that investigated the risk factors in 97 acoustic neuromas and 194 matched controls, no increased risk was found in regular phone use (OR = 0.67; 95 % CI 0.38–1.19) [20] (Table 3).

**Table 3:** Odds ratios (ORs) and 95 % confidence intervals (CIs) from case-control studies on acoustic neuromas and pituitary tumors

Study	No of Cases	No of Control	OR (95 % CI)
Acoustic neuroma			
Pettersson et al. 2014 [27]	451	710	1.18 (0.88–1.59)
Hardell et al. 2013 [24]	316	3.530	1.6 (1.2–2.2)
Schlehofer et al. 2007 [21]	97	194	0.67 (0.38–1.19)
Schoemaker et al. 2005 [25]	678	3.553	0.9 (0.7–1.1)
Pituitary Tumors			
Shrestha et al. 2015 [22]	80	240	0.39 (0.21–0.72)
Schoemaker et al. 2009 [26]	291	630	0.9 (0.7–1.3)
Takebayashi et al. 2008 [23]	102	683	0.90 (0.50–1.61)

#### Pituitary tumors

Regarding the relationship between development of pituitary tumors and mobile phone use, a recent case-control study that included 80 cases and 240 matched controls revealed no increase in pituitary tumor risk even after 10 or more years of usage. The risk was not influenced by call’s duration, hours of usage or cumulative number of calls. No difference was found for analog and digital phones [22]. A previous study that included 88 gliomas, 132 meningiomas, 102 pituitary adenomas and 683 individually matched controls evaluated the SAR inside the tumor. All maximal SAR values were below the levels at which thermal effects occurs. The results showed no overall increase in OR and there was no significant trend towards an increasing OR in relation to SAR [23] (Table 3).

## Conclusion

Although some small studies have showed a connection between intracranial tumors occurrence and mobile phone usage, this effect was not verified in larger series. The fact that some studies showed a reduced cancer risk, from biological point of view is difficult to believe that microwave exposure prevent brain tumors, thus possible metholodological errors in these studies should be sought. Furthermore, random errors or selection bias cannot be excluded in these studies [31]. Nevertheless, there was some evidence to suggest a connection between heavy mobile phone use and increased risk for brain tumor occurrence, especially for gliomas. Nevertheless, further studies are needed to study the possibility of long term use and gliomas. For meningiomas, acoustic neuromas and pituitary tumors the results are inconclusive. Thus, there is certainly a need for more studies and continuous surveillance.

## Abbreviations

CI: confidence interval
OR: odds ratio
RF: radiofrequency
SAR: specific absorption rate
WHO: World Health Organization
